# Out-of-pocket costs and time spent attending antenatal care services: a case study of pregnant women in selected rural communities in Zinder, Niger

**DOI:** 10.1186/s12913-020-06027-2

**Published:** 2021-01-08

**Authors:** Césaire T. Ouédraogo, Stephen A. Vosti, K. Ryan Wessells, Charles D. Arnold, M. Thierno Faye, Sonja Y. Hess

**Affiliations:** 1 Department of Nutrition, Institute for Global Nutrition, University of California, One Shields Ave, Davis, CA 95616 USA; 2 Department of Agricultural and Resource Economics, University of California, Davis, CA USA; 3 Helen Keller International, Niamey, Niger

**Keywords:** Out-of-pocket costs, Antenatal care, Opportunity cost of time, Pregnancy, Niger

## Abstract

**Background:**

Despite an official policy of exemption from health care costs, pregnant women in Niger still face some out-of-pocket costs (OPC) in addition to time costs when they attend antenatal care (ANC) services. We aimed to: 1) assess the OPC for pregnant woman attending ANC, 2) estimate the time spent to attend ANC and the opportunity cost of that time, and 3) assess how OPC and time spent to attend ANC affected ANC attendance.

**Methods:**

Data were obtained from a quasi-experimental descriptive study carried out in the region of Zinder, Niger, which compared pre- and post-intervention cohorts of pregnant women (*n* = 1736 women who reported attending ANC during their current pregnancy). An ANC attendance score was developed to describe the timing of ANC attendance in regard to the WHO recommendation of attending 4 ANC sessions. OPC and time spent were evaluated separately for associations with ANC attendance using Spearman correlations.

**Results:**

The mean (±SD) age of pregnant women was 25.0 ± 6.4 yr, 19.0% were ≤ 19 yr and 99.7% were in their second or third trimester of gestation at the time of the interview. Among those who were > 13 weeks and > 27 weeks of gestation, 4.0 and 74.4% had attended ANC during their first and second trimesters, respectively. The median (1st quartile (Q1), 3rd quartile (Q3)) ANC score was 0 (− 1, 0), reflecting that the majority of women failed to follow the WHO recommendation. More than half of the women (72.5%) experienced OPC related to ANC. The majority of women (> 80%) reported spending ~ 3 h for an ANC visit, including travel and waiting time. Time spent to attend ANC was not associated with ANC attendance score. Women who experienced OPC, and those who received iron folic acid (IFA) or long-lasting insecticide-treated bednets during an ANC visit, were more likely to have a higher ANC attendance score compared to those who did not.

**Conclusion:**

OPC and time spent were not identified as barriers to ANC visits, and IFA and long-lasting insecticide-treated bednets distribution could be used to motivate pregnant women to attend ANC.

**Trial registration:**

The NiMaNu project was registered at www.clinicaltrials.gov as NCT01832688. Registered 16 April 2013.

**Supplementary Information:**

The online version contains supplementary material available at 10.1186/s12913-020-06027-2.

## Background

Niger achieved a substantial reduction in neonatal mortality rate between 1990 and 2017, which decreased from 54 to 26 deaths per 1000 live births [1]. Although maternal mortality remains one of the highest in the world, maternal deaths per 100,000 live births also decreased, from 873 in 1990 to 553 maternal deaths in 2016 [2]. Numerous actions taken by the government of Niger that reduced socioeconomic inequities [2, 3], including an official policy established in 2006 that exempted pregnant women and children under 5 years of age from health care costs, may have contributed to this achievement [4]. For women, the official exemption from health care costs includes free access to family planning, routine antenatal care (ANC), caesarean section, and treatment of gynecologic cancers [4, 5]. For the routine ANC, the official exemption includes governmentally covered costs for the antenatal health booklet, consultations, intermittent preventive malaria treatment with sulfadoxine-pyrimethamine, iron folic acid (IFA) supplements, laboratory tests (glucose, albumin and infections), ultrasounds and consumable items necessary for the provision of these services [5].

Prior to 2016, the World Health Organization (WHO) recommended at least four ANC visits for uncomplicated pregnancies with the first ANC visit occurring before the 12th week of gestation, the second visit around 26 weeks, the third around 32 weeks and the fourth between 36 and 38 weeks of gestation [6]. The primary goal of ANC is to establish regular contact with pregnant women such that pre- and post-natal complications may be reduced. ANC consultations are conducted by health professionals and involve the delivery of routine services including review of medical history, detection and prevention of diseases, assessment of dangers signs and complications readiness, health education and birth preparedness. In Niger some progress has been made regarding ANC attendance over the past two decades. For example, from the 2006 to the 2012 Demographic and Health surveys (DHS) in Niger, the proportion of women who reported seeking any ANC from a qualified provider increased from 46 to 83% [7, 8]. However, even in the 2012 survey, after the free health care access to ANC had been introduced, only 33% of women reported attending at least four visits and 22% reported early ANC attendance (defined by the DHS survey as before 16 weeks gestation) during their most recent pregnancy [8].

Despite the official exemption from health care costs, pregnant women in Niger and elsewhere still face some out-of-pocket costs (OPCs) when they attend health services [9–13]. Previous studies found that payments were related to direct service (e.g., registration, consultation, laboratory examination, medications) or indirect costs such transportation or other costs. In addition, the time spent traveling to and from the ANC clinic and the time spent at a health center have cost implications for the women and/or her household [14, 15]. Systematic reviews of factors affecting utilization of ANC found associations with rural/urban residence, distance from health care facility, maternal age, parity, number of living children, women’s and husbands’ education, marital status, socioeconomic status, previous history of obstetric complications, women’s health knowledge, awareness and attitude, support from spouse or partner, quality of care and mass media exposure, cost for service and transportation [16–18]. There is limited information available about the relationship between ANC attendance and OPC, total time spent traveling and attending ANC, and opportunity cost of time to attend ANC in Niger, but previous studies conducted in Ethiopia, Tanzania, Bangladesh, and Pakistan have shown that higher OPC and greater travel time were associated with decreased maternal health care seeking [11, 19, 20].

In the context of the NiMaNu project, the present study aims to: 1) assess the OPC of pregnant woman associated with attending ANC, 2) estimate the total time spent and the opportunity cost of the time spent traveling to, waiting for and receiving ANC, and 3) assess how maternal and household characteristics, and OPC, total time spent and the opportunity cost of time for ANC visits affect ANC attendance.

## Methods

### Study design and participants

The Niger Maternal Nutrition (NiMaNu) project was designed as a quasi-experimental descriptive study conducted in the Zinder region of Niger. This project consisted of a baseline survey, a programmatic intervention and an endline survey, as described in detail elsewhere [21–24]. This project aimed to improve ANC services through programmatic interventions and used a multi-stage clustered sampling design to compare pre-and post-intervention cohorts of pregnant women. The aspects of the multi-level intervention were based on formative research [21] and consisted of community-based behavior change communication activities in each selected village, provision of supplies and essential drugs (including IFA supplements), and quality improvement activities at selected IHC. The primary outcomes of the main NiMaNu study were attending an adequate number of ANC consultations for gestational age (GA), reported adherence to recommended IFA supplementation, knowledge of the benefits of IFA, and prevalence of anemia [24].

In Niger, the health system in a district is organized at three levels: 1) health posts (HPs) provide very basic outpatient curative and preventive care and are staffed primarily by community health workers, 2) integrated health centers (IHCs) serve several villages, provide outpatient and inpatient care, including labor and delivery, and are staffed by nurses, midwives and sometimes physicians, and 3) a district hospital provides diagnostics services, outpatient and inpatient curative care, and caesarean sections, and receives patient referrals from IHCs [25, 26].

The project was implemented in two health districts in the Zinder region selected for convenience based on their seasonal accessibility, their distance to the town of Zinder and the small number of interventions with limited scope being implemented within their catchment area (i.e., to avoid overlapping interventions conducted in the same area). Within the two selected health districts, 18 rural IHCs out of 45 were selected based on convenience sampling and each IHC was randomly assigned to the order of enrollment. Within the catchment area of each IHC, the village in which the IHC was located was automatically included and nine additional villages were randomly selected according to the following scheme: first, among villages with a HP, one was randomly selected for inclusion. Second, among the remaining villages in the IHC catchment area, 4 villages ≤10 km and 4 villages > 10 km were randomly selected for inclusion. For both the baseline and endline surveys, pregnant women from the IHC and HP villages and the first two additional villages from the subsequent randomization were enrolled with a target of enrolling 16–20 women per village and a sample size of approximately 77 women per IHC. Participants were identified using a random walk method within each village [27], with the starting point randomly selected as either the mosque, market or primary school. A local guide accompanied the survey teams to help identify pregnant women during the random walk process. When the target number of women was not met in the first 4 villages of an IHC, women were included in sequential order from the remaining villages on the randomization list of that IHC. The enrollment of participants in the baseline survey was implemented continuously over a period of 18 months with approximatively one new IHC surveyed each month. Pregnant women irrespective of GA were eligible for study participation, provided they had resided in a participating village for at least 6 months prior to enrollment and had no plans to move out of the study area within the next 2 months. A woman was excluded if she had a severe illness warranting immediate hospital referral or was unable to provide consent due to impaired decision-making ability.

As soon as the baseline survey was completed in an IHC, a multi-level programmatic intervention was implemented in that IHC and its catchment area, and the endline survey was rolled out 6 months after the completion of the baseline survey (in the first 12 IHCs only). The programmatic intervention included community-based behavior change communication activities in each selected village, provision of supplies and essential drugs (including IFA supplements), and quality improvement activities at selected IHC [24].

### Data collection

Two separate cohorts of women participated in the baseline and the endline survey, respectively. The baseline survey started in March 2014 and the endline survey started in November 2014, and both continued until September 2015. In each survey, pregnant women were interviewed twice, approximately 1 month apart, to collect socio-demographic information, anthropometric measurements (weight, height, mid-upper arm circumference (MUAC), symphysis-fundal height) and knowledge, attitudes and practices (KAP) related to IFA supplementation, diet and antenatal care (e.g., ANC attendance in the current pregnancy, OPCs of attending ANC, and time spent to attend ANC). Village and health center level information was collected through interviews with health center staff and/or head of villages.

### Definition of variables

*Gestational age:* GA was assessed by asking each pregnant woman about the estimated number of months, lunar cycles and/or proximity to a religious or cultural event since she had her last menstrual period, if she already felt movements of the fetus in the current pregnancy and how long ago she first felt those movements, and two symphysis-fundal height measurements taken approximately 1 month apart. GA was then estimated based on the following obtained information: reported last menstrual period, time elapsed since the pregnant woman started to feel or perceive fetal movements, and the two symphysis-fundal height measurements. Methodological details are described elsewhere [28].

*ANC attendance score based on GA*: ANC attendance was assessed by asking each pregnant woman whether she attended ANC consultation at the IHC or HP or during outreach activities in the current pregnancy and the date she attended. The women’s responses were confirmed with their antenatal health booklet. Women were scored based on whether or not they had an ANC visit during each of four pregnancy intervals chosen based on the WHO ANC recommendation in place at the time of the present study [6]: first trimester: ≤13 weeks; second trimester: > 13 weeks to 27 weeks; first half of third trimester: > 27 weeks to 34 weeks; and second half of third trimester: > 34 weeks. For each interval, a woman received a score of 1 if she attended at least one ANC visit during that interval, and a score of − 1 if she did not attend ANC. If the woman’s fetal GA on the day of the interview was less than the end of the interval (i.e., woman had not yet completed that pregnancy interval) then she received a score of 0 for that interval, because she still had the opportunity to seek the recommended ANC in the respective period. The final ANC attendance score was calculated by adding these four scores together. Using a 3-value scale of − 1, 0 and 1 in each pregnancy interval differentiates those who attended a visit from those that did not, while also differentiating those whom may or may not attend their future visits. Additionally, we recalculated those scores according to the 2016 WHO recommendation of eight ANC contacts.

*Out-of-pocket costs per ANC visit attended during current pregnancy*: OPC costs were assessed by asking women to report how much she spent to pay for transportation, to and from the health center for each ANC visit attended and health care-related expenses for the consultation, medications, and laboratory tests. OPC were calculated for each ANC visit attended based on the pregnant women’s reported costs of transportation, for an ANC visit and health care-related expenses. The cost data were collected in West African CFA (XOF).

*Time spent per ANC visit attended during current pregnancy*: Time spent was assessed by asking each pregnant woman how much time she spent from the time she left her house until she returned and whether she used this time to accomplish other activities, if so what she did. Time spent was defined as the reported travel time to and from the site of ANC services plus the time spent in the health center while waiting for and receiving ANC and any related services. To calculate the opportunity cost of time, the variable “time spent to attend ANC”, which was collected as categorical variable, was converted into hours (i.e., a continuous variable) as follows: if a woman reported spending less than half day to attend ANC visit, she was considered having spent a mean (min, max) time of 3 (2, 4) hours; a half day was considered as a time of 6 (5, 7) hours; more than a half day, but less than 1 day, as a time of 9 (8, 10) hours; 1 day, a time of 12 (11, 13) hours; and 1 day and night, a time of 15 (14, 24) hours.

*Opportunity cost of time to attend ANC visit during current pregnancy*: The opportunity cost of time to attend each ANC visit was the opportunity cost of wages foregone by the woman due to her health center visit. More specifically, the opportunity cost of time was equal to time spent to attend ANC multiplied by an estimated hourly wage. Previous reports from Maradi and Tahoua, two neighboring regions to Zinder, found that women who engaged in income-generating activities (IGAs) earned incomes ranging from 223 to 292 XOF (i.e., 0.37 to 0.48 United States dollars (USD) per day in Maradi; 1USD = 601.1615 XOF based on exchange rate on 31 December 2015) [29] and 1000 to 1500 XOF (i.e., 1.66 to 2.50 USD) per day in Tahoua, respectively [30, 31]. Because information on wage rate is extremely limited and because the majority of pregnant women reported being housewives in the present study (i.e. not paid or self-paid), we estimated that women worked 11 to 13 h per day and their daily earnings were in the range of daily earnings of women engaged in IGAs in Maradi and Tahoua (i.e. 223 to 1500 XOF). Based on the daily earnings of women engaged in IGAs in Maradi and Tahoua and an assumed minimum of 11 work hours per day, the hourly wage estimated for pregnant women in the present study was 20 to 136 XOF per hour (i.e., 0.033 to 0.23 USD).

*Indicators of household socio-economic status (SES), food security, and dietary diversity:* Three proxy indices (household assets, household livestock, and housing quality) were developed to estimate SES using principal component analyses of available baseline household level indicators, including ownership of a set of assets, number of livestock, and home building materials, among others [32], as described in more detail previously [22]. Household food insecurity was assessed using the Household Food Insecurity Access (HFIA) categories [33]. Pregnant women’s dietary practices were assessed using a list-based food frequency questionnaire, and those who reported consuming at least five of ten defined food groups in the previous 24 h were considered to meet the Minimum Dietary Diversity for Women (MDD-W) [34].

### Sample size

The sample size of the main NiMaNu study was estimated to detect a difference of 10% in the prevalence of anemia as a primary outcome assuming a significance level of 0.05, power of 0.80, and a design effect of 2 [28]. The baseline and endline surveys enrolled different women with sample sizes of 1385 and 922, respectively. As previously presented [24], both cohorts of women differed in a few of their personal and household characteristics, such as the proportion of adolescent women, health facility delivery during last pregnancy, level of household food insecurity, principal occupation of the household head and season of enrolment in the survey (**Supplemental Table** **1**). The intervention did not affect reported ANC attendance [24]; in addition there was no association between the variable “impact” indicating if a participant was enrolled in the baseline or in the endline survey and their ANC score, and no modification of the relationship between ANC score and OPC by the variable indicating if a participant was enrolled in the baseline or in the endline survey. Therefore, all available data from the baseline and endline surveys were combined in the present analysis for a sample size of 1736 women (75.2% of women surveyed) who reported having attended at least one ANC visit in their current pregnancy. This sample size provides high precision in estimation. Specifically, we can estimate the ANC attendance prevalence with precision of ±3.5% (95% CI) and the ANC attendance score with 95% confidence interval (CI) of ±0.05 standard deviation (SD).

### Statistical analysis

Statistical analyses were performed using the SAS System, version 9.4 (SAS Institute Inc., Cary, NC, USA). A detailed statistical analysis plan is available online [28]. Descriptive analysis of initial characteristics of study participants was performed. Any OPC for ANC visits attended during current pregnancy (yes or no), mean OPC of attended ANC visits per woman, and mean time spent for ANC visits per woman were tested separately for associations with the ANC attendance score based on the 2002 and 2016 WHO recommendation of at least four ANC visits and eight ANC contacts, respectively in minimally adjusted models including GA (in weeks) using Spearman correlations and in covariate adjusted models using partial Spearman correlations. As additional sensitivity analysis, the same test for association were repeated stratified by survey cohort. The partial Spearman correlation technique is a non-parametric analog to the covariate adjustment used in linear regression models to control for confounding. For each woman we first considered three different assumptions regarding her time spent per ANC visit attended (i.e., a mean, a minimum and a maximum value) and each of these values was used in the analysis. There was no variability in the relationship between time spent and ANC attendance score. Therefore, we decided to use the mean time spent in the analysis. The mean ANC attendance score was compared via ANCOVA in the group of women who experienced no OPC and the group of women with any OPC. This was completed for both minimally adjusted and adjusted analyses. In the sub-sample of women who reported any ANC related cost, we performed a sensitivity analysis by looking at the relationship between ANC attendance score and specific OPCs for consultation, transportation and exam; and we also looked at the relationship between OPC expenses in private clinics and ANC attendance score.

Potential adjustment covariates were identified based on a literature review and background knowledge of the association of cost and time with ANC attendance [28], and were examined by performing bivariate analyses. These included individual characteristics (i.e., maternal age, education level, marital status, rank of the woman in a polygamous marriage, occupation, age at first pregnancy, number of pregnancies, number of living children, outcome of previous pregnancy (child born alive and still living, stillbirth and abortion), location of delivery during last pregnancy (health facility vs home), gestational age (in weeks), self-reported experience of any danger signs during the current pregnancy, and referral to health center because of low MUAC (< 23 cm) measured during enrollment), household level characteristics (i.e., household asset index, household livestock index and housing quality used as a proxy of household SES (with each index treated as binary variable i.e., above median vs at or below the median), household head’s education level, principal occupation of the household head, level of household food insecurity (HFIAS categories), receipt of food assistance (yes or no), environmental characteristics (season of enrollment), ANC visit characteristics (OPCs and time spent attending actual ANC visits), and receipt of IFA or insecticide-treated bed nets during ANC). Only covariates significantly associated with the ANC attendance score at a level of significance ≤0.1 were included in the covariate adjusted models.

## Results

### Characteristics of study site and population

**Fig. 1:**
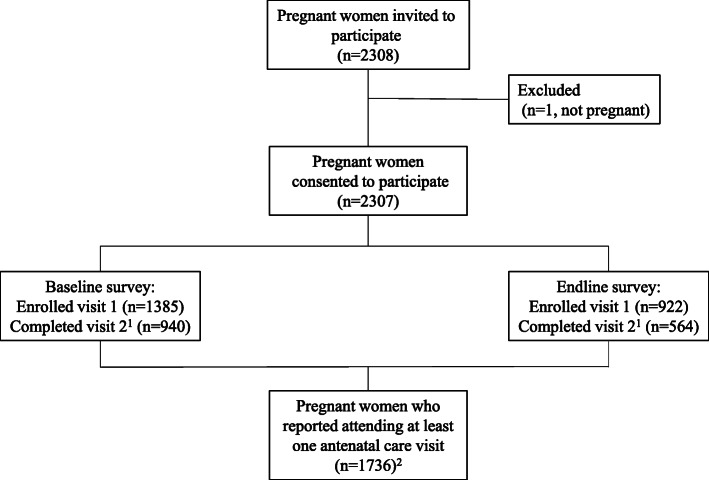
Flow chart of participants included in the out-of-pocket costs and time cost analysis ^1^Visit 1 and 2 were one month apart, attrition was due to birth (n=584), relocation (n=107), consent withdrawal (n=59), stillbirth (n=49) and maternal death (n=4) ; By either visit 1 or 2

In total, participants were enrolled from 92 villages into the main trial, with 24% of the villages situated on paved road (Table 1). Among the 1385 and 922 pregnant women enrolled in the baseline and endline surveys during the main trial respectively, a total of 1736 pregnant women reported having attended any ANC visit during their current pregnancy (Fig. 1). Compared to those who reported not having attended ANC in their current pregnancy, women who reported attending ANC visits were more likely to have been enrolled in the survey during the hot season, were more likely to be in their third trimester of gestation, and were more likely to have attended ANC during their previous pregnancy and delivered in a health facility (Table 2**)**. In addition, pregnant women who had attended ANC were more likely to have some formal education, to live in a household where the household head had some formal education and to be engaged in a non-farming occupation. Among those women who had attended at least one ANC in their current pregnancy, their mean age was 25.8 ± 6.4 years and 21.1% had some formal education. More than half were in their third trimester of gestation and 72.5% reported OPC associated with ANC visits (Table 2).

**Table 1:** Characteristics of the sampled villages in Zinder, Niger

Variables	n (%)
**Distance of the village from the paved road**	
Situated on the paved road	20 (23.8)
≤ 10 km from the paved road	32 (38.1)
> 10 km from the paved road	32 (38.1)
Missing data	8
**Number of traditional birth attendants serving the village**	
None	5 (6.0)
One	15 (18.1)
Two	42 (50.6)
Three or more	21 (25.3)
**Number of community health workers serving the village**	
None	20 (23.8)
One	18 (21.4)
Two	21 (25.0)
Three or more	25 (29.8)
**Number of integrated health centers serving the village**	
None	2 (2.4)
One	66 (78.6)
Two or more	16 (19.0)
**Number of health posts serving the village**	
None	33 (39.3)
One	35 (41.7)
Two or more	16 (19.0)
**At least one pharmacy in the village**	20 (23.8)
**A market in the village**	20 (23.8)
**Electricity in the village**	14 (17.3)
**At least one microcredit organization in the village**	20 (23.8)
**At least one health organization in the village**	18 (21.4)
**At least one women’s organization in the village**	42 (50.0)
**At least one husbands’ group in the village**	12 (14.3)

**Table 2:** Characteristics of pregnant women and their household

	Reported attending ANC^**a**^	Reported not attending ANC^**a**^	P-value
**Participants, n**	1736	560	
**Age**, years (mean ± SD)	25.8 ± 6.4	26.0 ± 6.4	0.86
**Adolescent (≤ 19 years)**	324 (19.0%)	94 (16.8%)	0.24
**Ethnicity**			
Hausa	1469 (84.7%)	482 (84.7%)	
Others	266 (15.3%)	87 (15.3%)	0.98
**Education**			
Any formal education	383 (22.1%)	103 (18.1%)	
No education	1352 (77.9%)	466 (81.9%)	0.04
**Principal occupation**			
Housewife	1420 (81.8%)	476 (83.7%)	
Non-Housewife	316 (18.2%)	93 (16.3%)	0.31
**Trimester**^**b**^			
First	5 (0.3%)	38 (6.7%)	
Second	550 (31.9%)	409 (72.4%)	
First half of third trimester	557 (33.4%)	67 (11.9%)	
Second half of third trimester	593 (34.4%)	51 (9.0%)	<.0001
**Obstetric history**			
Age at first pregnancy median, years (25th, 75th)	17.0 (16.0–18.0)	16.0 (16.0, 17.0)	0.62
(Min, max)	(11.0, 35.0)	(13.0, 38.0)	
**Gravidity**			
Multigravida	1487 (85.7%)	501 (88.1%)	
Primigravida	249 (14.3%)	68 (11.9%)	0.15
**Outcome of previous pregnancy**			
Child born alive, still living	1286 (86.5%)	428 (85.4%)	
Child born alive, has since died	138 (9.3%)	56 (11.2%)	
Still birth, miscarriage, abortion	63 (4.2%)	17 (3.4%)	0.35
**Attended any ANC during last pregnancy**	1383 (93.0%)	452 (90.2%)	0.04
**Attended at least 4 ANC during last pregnancy**	601 (40.4%)	175 (35.1%)	0.03
**Health facility delivery during last pregnancy**	580 (39.1%)	149 (29.8%)	0.0002
**Household level characteristics**			
**Household head’s education level**			
Any formal education	337 (22.8%)	66 (13.0%)	
No education	1144 (77.2%)	441 (87.0%)	<.0001
**Principal occupation of the household head**			
Farming related occupation	546 (31.8%)	237 (41.9%)	
Non-farming related occupation	1170 (68.2%)	328 (58.2%)	<.0001
**Levels of household food insecurity**			
Food secure	767 (44.2%)	222 (39.1%)	
Mildly food insecure	152 (8.8%)	50 (8.8%)	
Moderately food insecure	389 (22.4%)	141 (24.9%)	
Severely food insecure	427 (24.6%)	154 (27.2%)	0.19
**Season of enrollment**			
Lean, rain (June–September)	575 (33.1%)	201 (35.3%)	
Dry, post-harvest (October–February)	590 (34.0%)	221 (38.8%)	
Hot (March–May)	571 (32.9%)	147 (25.8%)	0.006

^a^*ANC* Antenatal care; ^b^Trimester at visit 1

### Distribution of ANC visits and ANC attendance score by GA

**Fig. 2:**
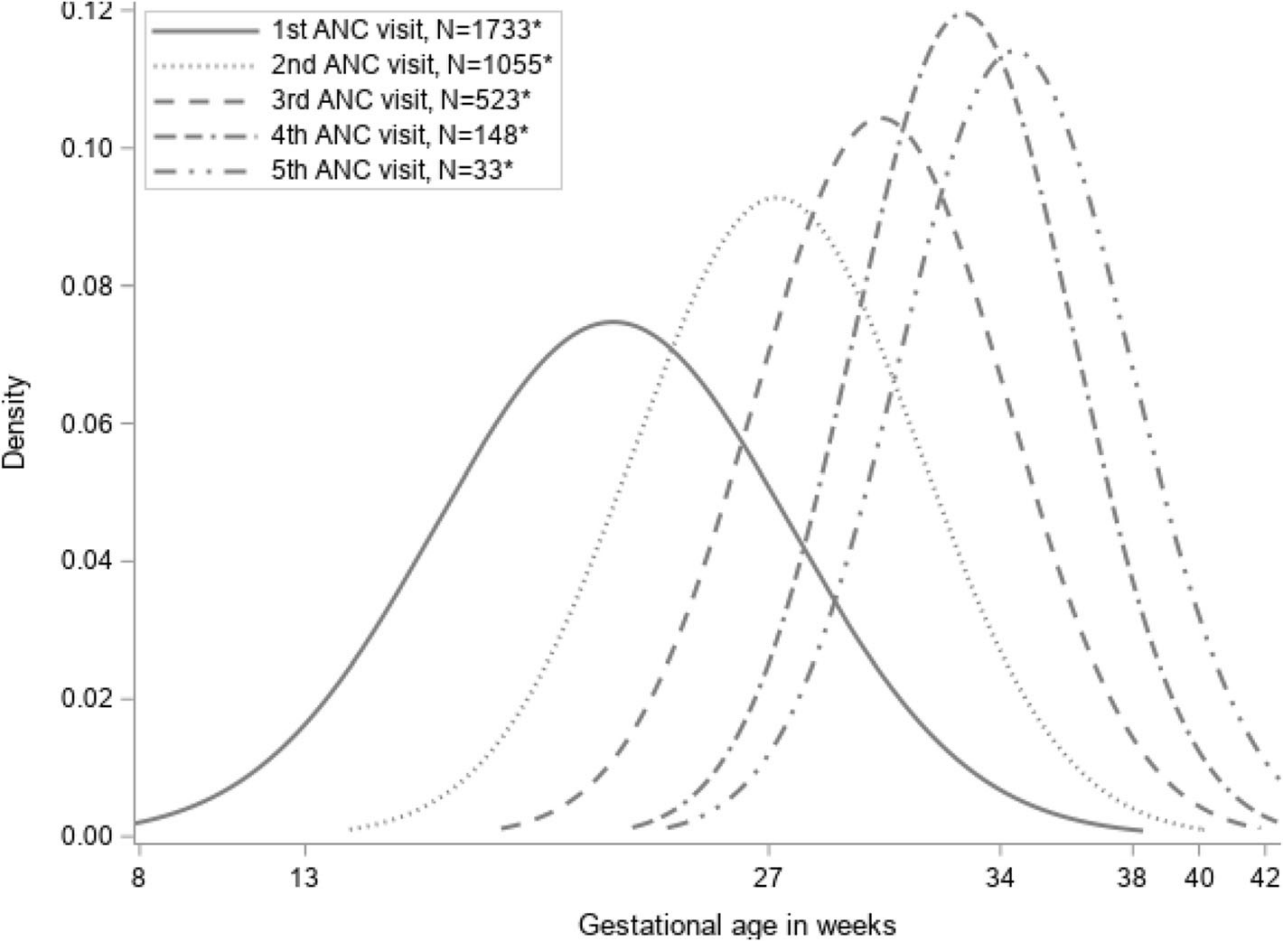
Smoothed distribution of antenatal care (ANC) visits by women’s gestational age from the first to the fifth ANC visit, assuming a normal distribution *Women were interviewed at any time of their pregnancy and had self-selected how many antenatal care (ANC) visits to attend and reported on sequential visits from earlier intervals, thus resulting in different available sample size per ANC visit

The majority of women sought ANC for the first time in their second trimester (Table 3). Among those who were > 13 weeks and > 27 weeks pregnant at the time of the interview, 4.0 and 74.4% had attended ANC during their first trimester and their second trimester, respectively **(**Fig. 2**)**.

**Table 3:** Timing of ANC attendance compared to 2002 WHO recommendation of 4 ANC visits per pregnancy ^a, b, c^

ANC attendance compared to WHO recommendation^**d**^	ANC attendance scoring criterion	First trimester	Second trimester	First half of third trimester	Second half of third trimester
Attended	1	68 (4.0%)	1127 (65.8%)	487 (28.4%)	14 (0.8%)
Failed to attend	-1	1644 (95.9%)	348 (20.3%)	76 (4.4%)	6 (0.4%)
Not applicable^e^	0	2 (0.1%)	239 (13.9%)	1151 (67.1%)	1694 (98.8%)

^a^*ANC* Antenatal care; *WHO* World Health Organization

^b^Sample size: *n* = 1714;

^c^Trimesters were defined as: first trimester, ≤13 weeks; second trimester: > 13 weeks to 27 weeks; first half of third trimester: > 27 weeks day to 34 weeks; and second half of third trimester: > 34 weeks;

^d^Until 2016, WHO recommended at least four ANC visits for uncomplicated pregnancies with the first ANC visit occurring before the 12^th^ week of gestation, the second around 26 weeks, the third around 32 weeks and the fourth visit between 36 and 38 of gestation;

^e^Not applicable because women had not yet completed respective trimester period

Because the ANC attendance score depends on the GA at the time of the interview, the minimum acceptable attendance score based on the four ANC visits could range from 0 to 3 and the maximum acceptable score could range from 1 to 4. The median (1st quartile (Q1), 3rd quartile (Q3)) ANC attendance score based on the four ANC visits was constant from the first trimester to the first half of the third trimester of gestation (0 (− 1, 0)) and increased to 1 (− 1, 1) in the second half of the third trimester of gestation (Table 4**)**. There was no significant difference between the cohort of women participating in the baseline and the endline survey (**Supplemental Tables** **2****a and** **2****b)**. Using the ANC attendance score based on the eight ANC contacts, the minimum acceptable attendance score could range from 0 to 7 and the maximum acceptable score from 1 to 8. The median (Q1, Q3) ANC attendance score was constant from the first trimester to the first half of the second trimester of gestation (0 (− 1, 0)), decreased over the rest of second trimester and increased to (1 (− 0.5, 2)) at the end of the third trimester (**Supplemental Table** **3****a**). There was no significant difference between the cohort of women participating in the baseline and the endline survey in ANC score based on eight ANC visits (**Supplemental Tables** **3****b and** **3****c**).

**Table 4:** ANC attendance score based on 4 ANC visits by gestational age (trimester) of pregnancy^a, b^

	First trimester	Second trimester	First half of third trimester	Second half of third trimester
Minimum acceptable ANC attendance score	0	1	2	3
Maximum possible ANC attendance score	1	2	3	4
Median (Q1, Q3) attendance score^c^	0 (−1, 0)	0 (−1, 0)	0 (−1, 0)	1 (− 1, 1)

^a^*ANC* Antenatal care;

^b^Trimesters were defined as: first trimester, ≤13 weeks; second trimester: > 13 weeks to 27 weeks; first half of third trimester: > 27 weeks day to 34 weeks; and second half of third trimester: > 34 weeks

^c^Median (1st quartile (Q1), 3rd quartile (Q3))

### Time spent, type of transportation, and opportunity cost of time of attending ANC

At each ANC visit, the majority of pregnant women (83.8–86.2%) spent less than half a day (~ 3 h) to attend an ANC session, and only 10.7–13.5% and 13.8–16.2% of women reported having spent half a day and greater than half a day respectively (Table 5). Among those who spent less than half a day, most women (88.5%) reported having walked to attend ANC. The median opportunity cost of time was estimated as ranging from 60 to 408 XOF (i.e., 0.10 to 0.68 USD) for a minimum of 11 h worked and based on an hourly wage ranging from 20 to 136 XOF (i.e., 0.033 to 0.23 USD). Assuming a maximum of 13 h worked and based on the aforementioned range of daily earning, the estimated median opportunity cost of time ranged from 51 to 345 XOF (i.e., 0.08 to 0.57 USD).

**Table 5:** Reported time spent for sequential ANC visits by pregnant women^a^

Time spent	Percentage of women reporting for each ANC visit attended				
	First	Second	Third	Fourth	Fifth
N^b^	1733	1055	523	148	33
Less than half a day	1463 (84.4%)	897 (85.0%)	449 (86.2%)	124 (83.8%)	27 (84.4%)
Half a day	185 (10.7%)	109 (10.3%)	54 (10.4%)	20 (13.5%)	4 (12.5%)
More than half day	81 (4.7%)	43 (4.1%)	18 (3.4%)	4 (2.7%)	1 (3.1%)

^a^*ANC* Antenatal care

^b^Women were interviewed at any time of pregnancy, had self-selected number of ANC visits to attend and reported on sequential visits from earlier intervals, thus resulting in different available sample size per ANC visit

### Out-of-pocket costs per ANC visit by types of health center

Among women who attended ANC, most women (71.6%) experienced expenses related to ANC at the first ANC visit and 15.9% had OPC at the second visit (Table 6). Only a small proportion of women reported spending money during their subsequent visits. Among women who attended ANC and reported OPC, the majority visited IHC and HP (i.e., public first-level facilities) for all ANC visits. Median OPC were fairly constant across visits among women who attended ANC at IHC or HP, and ranged from 200 to 300 XOF (Table 6). At the district hospital, reference maternity hospital, and private clinics the median OPC were substantially higher. The majority of OPC dispensed by women were related to consultation expenses, primarily occurring at the first visit. Few women (< 15%) reported OPCs for exams, medications and transportation expenses across visits **(**Table 7**)**.

**Table 6:** Median (Q1, Q3) out-of-pocket costs for sequential antenatal care visits by type of health care facility ^a, b^

	Antenatal care visits				
	First	Second	Third	Fourth	Fifth
N, total	1733	1055	523	148	33
N (%), reporting no costs^c^	490 (28.4%)	879 (83.3%)	448 (85.7%)	134 (90.5%)	30 (90.9%)
N (%), reporting some OPC^c^	1240 (71.6%)	168 (15.9%)	69 (13.3%)	13 (8.8%)	3 (9.1%)
**IHC & HP**^**d**^					
N (%)^e^	1209 (97.5%)	149 (88.7%)	55 (79.7%)	10 (76.9%)	3 (100%)
OPC at IHC & HP	200 (100, 300)	250 (200, 700)	300 (250, 900)	250 (250, 400)	250 (200, 750)
**District hospital & reference clinic**					
N (%)^e^	21 (1.7%)	5 (3.0%)	2 (2.9%)	0	0
OPC at district hospitals	350 (100, 2500)	1749 (1200, 4250)	950 (700, 1200)	–	–
**Private clinics**					
N (%)^e^	6 (0.5%)	13 (7.7%)	12 (17.4%)	3 (23.1%)	0
OPC at private clinics	2750 (800, 3800)	900 (600, 1100)	900 (650, 3000)	700 (600, 1150)	–

^a^Median (1st quartile (Q1), 3rd quartile (Q3)) cost among pregnant who spent money during antenatal care visits

^b^Costs shown in West African CFA (XOF), 1USD = 601.1615 XOF based on exchange rate on 31 December 2015

^c^Percentages do not sum to 100% because of missing values

^d^IHC, Integrated health center; HP, Health post; OPC, out-of-pocket costs

^e^Women were interviewed at any time of pregnancy and had self-selected how many ANC visits to attend and reported on sequential visits from earlier intervals, thus resulting in different available sample size per ANC visit

**Table 7:** Reported out-of-pocket costs by sequential antenatal care visit among pregnant women^a,b^

Antenatal care visit										
	**First**		**Second**		**Third**		**Fourth**		**Fifth**	
	n/N (%)^c^	Median (Q1, Q3) cost among “spenders”^d^	n/N (%)^c^	Median (Q1, Q3) cost among “spenders”^d^	n/N (%)^c^	Median (Q1, Q3) cost among “spenders”^d^	n/N (%)^c^	Median (Q1, Q3) cost among “spenders”^d^	n/N (%)^c^	Median (Q1, Q3) cost among “spenders”^d^
**Overall out-of-pocket costs per visit**	1240/1733 (71.6%)	200 (100, 300)	168/1055 (15.9%)	300 (200, 900)	69/523 (13.2%)	500 (250, 900)	13/148 (8.8%)	300 (250, 600)	3/33 (9.1%)	250 (200, 750)
**Consultation**	1085/1733 (62.6%)	200 (100, 200)	51/1055 (4.8%)	200 (100, 900)	15/523 (2.9%)	900 (250, 1000)	3/148 (2.0%)	250 (100, 250)	0	–
**Exams**	253/1733 (14.6%)	250 (200, 350)	69/1055 (6.5%)	250 (250, 250)	30/523 (5.7%)	250 (250, 250)	8/148 (5.4%)	250 (200, 250)	2/33 (6.1%)	225 (200, 250)
**Medications**	77/1733 (4.4%)	1250 (600, 2000)	21/1055 (2.0%)	1200 (750, 2000)	7/523 (1.3%)	1000 (750, 1600)	1/148 (0.7%)	400 (400, 400)	1/33 (3.0%)	750 (750, 750)
**Transportation**	142/1733 (8.2%)	300 (200, 500)	57/1055 (5.4%)	300 (200, 500)	32/523 (6.1%)	350 (225, 500)	5/148 (3.4%)	500 (400, 500)	0	–

^a^Proportion of pregnant women who spent money during antenatal care visits: 1255 (72.4%)

^b^Costs shown in West African CFA (XOF), 1USD = 601.1615 XOF based on exchange rate on 31 December 2015

^c^n = Participants who spent any amount of money during each ANC visit; N = Number of pregnant women who attended each ANC session regardless if they spent or not some money durning an antental care visit.

^d^Median (1st quartile (Q1), 3rd quartile (Q3))

### Relationship between ANC attendance score and maternal and household characteristics

Table 8 shows the bivariate analyses in which we modelled unadjusted associations between ANC attendance score and each of several maternal and household characteristics. As expected, with increasing GA the ANC attendance score increased (sample Spearman ρ coefficient, *p*-value: 0. 32, < 0.0001). A higher ANC attendance score was also more likely among women who had delivered in a health facility during their last pregnancy, among those who reported recent receipt of food assistance, among women who reported receiving an insecticide-treated bednet during their current pregnancy, among those who received IFA supplements during their current pregnancy, among those with higher SES and among women who lived in a household where the household head had formal education. Higher ANC attendance scores were less likely among women who lived in households where the household heads identified themselves as farmers and among women who were interviewed in the study during the lean and rainy seasons compared to those interviewed during the hot season. When the two survey cohorts of participants were analyzed individually and regardless of the scale of ANC attendance score (based on 4 ANC or 8 contacts), health facility delivery during their last pregnancy, reported recent receipt of food assistance, receipt of IFA supplements during the current pregnancy, and higher SES (i.e., high household asset index and housing quality) were associated with a higher ANC attendance score **(Supplemental Tables** **4****and** **5****).**

**Table 8:** Maternal and household characteristics associated with ANC attendance score based the 2002 and 2016 WHO recommendations to attend at least four or eight ANC visits per pregnancy (n = 1736)^a, b^

	ANC attendance score based on four ANC visits^c^		ANC attendance score based on eight ANC contacts^d^	
	Spearman correlation or mean difference (95% CI)	P-value^**e**^	Spearman correlation or mean difference (95% CI)	P-value^**e**^
**Age, years**	0.04	0.12	0.003	0.90
**Adolescent**				
Yes	−0.09 (− 0.21, 0.04)	0.17	− 0.08 (− 0.28, 0.11)	0.40
No	Ref		Ref	
**Education level**				
Any formal education	0.002 (−0.11, 0.12)	0.97	0.01 (−0.17, 0.19)	0.88
No education	Ref		Ref	
**Rank of woman in polygamous marriage**	0.03	0.47	0.03	0.47
**Principal occupation**				
Housewife	0.005 (−0.12, 0.13)	0.94	−0.16 (0.36, 0.03)	0.10
Non-housewife	Ref		Ref	
**Age at first pregnancy**	0.009	0.72	0.009	0.70
**Number of pregnancies**	0.02	0.40	−0.008	0.76
**Number of living children**	0.04	0.17	0.008	0.75
**Outcome of previous pregnancy**				
Child born alive, still living	−0.04 (− 0.20, 0.11)	0.61	− 0.13 (− 0.37, 0.10)	0.26
Child not born alive or born alive and has since died	Ref		Ref	
**Health facility delivery in previous pregnancy**	0.15 (0.04, 0.25)	0.006	0.28 (0.11, 0.44)	0.0008
**Gestational age (weeks)**	0.32	<.0001	0.03	0.24
**Reported experiencing any danger signs in current pregnancy**	0.02 (−0.18, 0.22)	0.83	0.006 (− 0.30, 0.32)	0.97
**Referred to health center because of undernutrition at visit 1**^**f**^	−0.03 (− 0.14, 0.08)	0.56	0.05 (− 0.12, 0.22)	0.58
**Reported receiving food assistance the month before the interview**	0.33 (0.18, 0.48)	<.0001	0.46 (0.23, 0.70)	0.0001
**Received mosquito net**	0.18 (0.06, 0.31)	0.005	−0.009 (− 0.21, 0.19)	0.92
**Has received iron folic acid supplements**	0.72 (0.62, 0.82)	<.0001	0.84 (0.69, 1.0003)	<.0001
**Household asset index**				
Above median	0.19 (0.10, 0.29)	<.0001	0.22 (0.07, 0.37)	0.005
At or below the median	Ref		Ref	
**Household livestock index**				
Above median	−0.002 (−0.10, 0.09)	0.97	0.03 (−0.12, 0.18)	0.68
At or below the median	Ref		Ref	
**Housing quality**				
Above median	0.10 (0.01, 0.20)	0.03	0.02 (−0.13, 0.17)	0.77
At or below the median	Ref		Ref	
**Household head’s education level**				
Any formal education	0.15 (0.02, 0.27)	0.02	0.23 (0.003, 0.42)	0.02
No education	Ref		Ref	
**Principal occupation of the household head**				
Farming related occupation	−0.13 (−0.23, −0.02)	0.01	−0.21 (− 0.38, − 0.05)	0.01
Non-farming related occupation	Ref		Ref	
**Levels of household food insecurity**				
Food secure	0.11 (−0.01, 0.23)	0.20	0.12 (−0.06, 0.31)	0.48
Mildly food insecure	0.17 (−0.02, 0.35)		0.10 (−0.20, 0.39)	
Moderately food insecure	0.10 (−0.04, 0.24)		0.16 (−0.06, 0.38)	
Severely food insecure	Ref		Ref	
**Season of enrollment**				
Lean, rain (June–September)	−0.14 (−0.26, − 0.02)	0.06	−.28 (− 0.47, − 0.10)	0.01
Dry, post-harvest (October–February)	−0.08 (− 0.20, 0.03)		−0.16 (− 0.35, 0.02)	
Hot (March–May)	Ref		Ref	

^a^*ANC* Antenatal care

^b^Only women who attended ANC visits

^c^Until 2016, WHO recommended at least four ANC visits for uncomplicated pregnancies with the first ANC visit occurring before the12^th^ week of gestation, the second around 26 weeks, the third around 32 weeks and the fourth visit between 36 and 38 of gestation;

^d^In 2016, WHO recommended at least eight ANC contacts with the first ANC contact occurring before the12^th^ week of gestation, the second around 20 weeks, the third around 26 weeks, the fourth around 30 weeks, the fifth around 34 weeks, the sixth around 36, the seventh around 38 weeks, and the eighth around 40 weeks of gestation;

^e^*P*-values are from Spearman correlation (for continuous variable) and t-test/omnibus ANOVA F-test (for categorical variables)

^f^Undernutrition defined as mid-upper arm circumference (MUAC) < 23 cm

### Effects of out-of-pocket costs and time spent to attend ANC visit on ANC attendance score

Pregnant women who spent any money to attend ANC sessions were more likely to have a higher ANC attendance score compared to those who did not (Table 9). This was true in both minimally adjusted analysis and when adjusted for the aforementioned significant covariates, although these associations were only significantly with the ANC score based on four ANC visits (**Supplemental Tables** **6****and** **7****)**. In the sensitivity analysis, women who spent money at the first visit were more likely to attend a second ANC visit (odds ratio (95%CI): 1.26 (1.02, 1.56)) but there was no relationship between OPC spent at the second, third or subsequent ANC visits. There was an association between ANC attendance score and OPCs for consultations, medications and exams. Women who paid any money for consultations, exams and medications were also more likely to have a higher ANC attendance score. When data is limited to women who paid to attend ANC, OPC was associated with a decrease in ANC attendance score based on eight ANC contacts. In contrast, the time spent to attend ANC was not associated with ANC attendance score regardless of the scale of ANC attendance score used (Table 9**).** These results were consistent also when the baseline and endline survey cohort were analyzed separately **(Supplemental Tables 6 and 7**).

**Table 9:** Association between out-of-pocket costs and time cost of attending ANC visit and ANC attendance score ^**a**^

	ANC attendance score based on four ANC visits				ANC attendance score based on eight ANC contacts			
	Minimally adjusted Spearman correlation or mean difference (95% CI)^**b**^	P-value	Adjusted Spearman correlation or mean difference (95% CI)^**c**^	P-value	Minimally adjusted Spearman correlation or mean difference (95% CI)^**b**^	P-value	Adjusted Spearman correlation or mean difference (95% CI)^**d**^	P-value
**Any out-of-pocket costs spent per ANC visit**								
Yes	0.27 (0.16, 0.38)	< 0.0001	0.22 (0.10, 0.35)	0.005	0.26 (0.08, 0.44)	0.005	0.20 (− 0.01, 0.40)	0.06
No	Ref		Ref		Ref		Ref	
**Mean out-of-pocket costs (XOF)** ^**e,f**^	0.002	0.921	0.021	0.531	−0.07	0.005	−0.09	0.002
**Mean time spent (hours)**	0.02	0.46	0.05	0.08	−0.003	0.90	0.02	0.45

^a^ANC, Antenatal care

^b^Controlling for only gestational age (in weeks),

^c^Controlling for only maternal and household characteristics significantly associated with the outcomes at a level of significance of 0.1 in bivariate analysis (i.e., for gestational age (in weeks), health facility delivery in previous pregnancy, reported receiving receipt of food assistance the month before the interview, reported receipt of insecticide-treated bednet, and IFA supplement through ANC, indicators of household SES (housing quality and household asset index), household head’s education level, principal occupation of the household head, season of enrollment in the study;

^d^Controlling for only maternal and household characteristics significantly associated with the outcomes at a level of significance of 0.1 in bivariate analysis (i.e., for gestational age (in weeks), principal occupation of the participant, health facility delivery in previous pregnancy, reported receiving receipt of food assistance the month before the interview, reported receipt of IFA supplement through ANC, indicators of household SES (household asset index), household head’s education level, principal occupation of the household head, season of enrollment in the study;

^e^In West African CFA (XOF), 1USD = 601.1615 XOF based on exchange rate on 31 December 2015

^f^Among those participants who paid out-of-pocket costs (*n* = 1254)

## Discussion

The present study showed that the majority of women sought ANC for the first time in their second trimester. The median ANC attendance score was 0 for most women who were in their third trimester of pregnancy, reflecting that the majority of women failed to follow the WHO recommendations of four ANC visits per pregnancy [6], as was recommended at the time of the study implementation. This finding was consistent when the ANC attendance score was based on the WHO recommendation of eight contacts. The majority of women spent less than half a day seeking ANC including travel, waiting and consultation time. In spite of the governmental exemption from health care costs for ANC, most women experienced expenses related to ANC, primarily incurred at the first ANC visit. However, any OPC was associated with a higher ANC attendance score, suggesting that women who spent some money were more likely to attend a subsequent ANC visit.

In the present study, few pregnant women (4.0%) attended ANC during the first trimester as recommended by the 2002 WHO recommendation [6]. In contrast, in the Niger 2012 DHS, 22% of pregnant women reported having attended their first ANC visit before 4 months gestation during their most recent past pregnancy [8]. The difference in results may be due to different cutoffs between surveys (< 16 weeks vs. ≤13 weeks in the DHS and the present survey, respectively). However, when we applied the same cut-off as the DHS in the present study, only 12.3% women reported their first ANC in their first 4 months of pregnancy, which was still substantially lower than the 22% at national level in the 2012 DHS. A reason for this discrepancy may be that the DHS relied on women’s self-determination of GA, whereas we attempted to assess GA based on reported last menstrual period, time elapsed since fetal movements were felt, and two fundal height measurements taken approximately 1 month apart [28]. Moreover, we verified the date of the ANC visit with the antenatal health booklet, which was not done in the DHS.

It is well established that women in low-income countries often attend their first ANC later than the first trimester. A systematic review on the regional and global level and trends of coverage of early ANC found that the estimated coverage of early antenatal care visits (< 12 week gestation) was 24.0% (95% uncertainty interval 21.7–26.5) in low-income countries [35]. We found that the majority of women sought ANC for the first time in their second trimester. Considering that the timing of initiation of the first ANC visit is considered important for ensuring optimal care and health outcomes for women and their offspring [35], and that the region of Zinder has the lowest health care coverage rate in the country [36], additional efforts should be made to encourage women in Zinder to seek ANC early in their pregnancy.

We have developed a novel ANC attendance score to describe the timing of ANC attendance in regard to the WHO recommendations of four ANC visits per pregnancy, as was recommended at the time of the study implementation. To explore the relevance of the ANC score when compared to the more recent WHO recommendation, we also explored how the ANC score performed when compared to eight contacts per pregnancy. The ANC score as calculated is not a count of ANC visits but it is attempting to capture more how closely a woman follows recommendations. This new ANC score can be a promising tool to evaluate ANC attendance of a population, which will require further validation. In the present study, the median ANC score based on at least four ANC visits was 0, suggesting that the majority of women did not meet the WHO recommendation. This was consistent when the ANC score based on the more recent WHO recommendation was used and also when the baseline and endline survey cohort were analyzed separately. This was because only few women attended their first ANC in early pregnancy and nearly half of women attended ANC for the first time by the end of their second trimester. However, the ANC score, as used in the present study, had an important limitation. Because women participating in this study were interviewed at different points of time during their pregnancies, a zero was assigned to incomplete pregnancy periods when calculating the ANC score. For example, more than half of the women in their first half of third trimester did not complete this respective period and therefore could not be identified as having attended ANC or not. Thus, some women were penalized by imputing zeros for their future visits because they had not yet completed their respective period. This may have led to a negative bias in the relationship between ANC attendance score and the predictors. However, this bias was reduced by adjusting for GA age in the minimally adjusted and fully adjusted models. Although we had asked women if they planned to attend future ANC in the remainder of their pregnancy, we decided against imputing the ANC score because such predictions are not reliable and are suspect to social desirability bias. However, the score may be more accurate if applied for post-partum women.

Among those who attended ANC, the majority of pregnant women (~ 80%) spent less than half a day to attend an ANC session regardless of the season and mode of transportation, even though most pregnant women reported having walked to attend ANC. In Rwanda, the amount of time to attend ANC, including walking time to and from the health center, was on average between four and five hours [15]. In contrast, in Malawi, a study has shown that pregnant women spent much more time (i.e. mean time of 7.7 h (SD 4.3)) to attend ANC [37]. Because the majority of women walked in these studies like women in the present study, the difference in time spent to attend ANC may be due to differences in the physical distance and/or accessibility of the health facility, and the waiting time spent at the health center, which may depend on the day of the consultation and the number of health workers in the health center. For example from 2013 to 2015, the ratio of midwife to women of reproductive age and nurse to population significantly improved in Niger [38, 39].

For the majority of pregnant women in the present study, we estimated the median opportunity cost of time per ANC visit and per women to range from 60 to 408 XOF (i.e., 0.10 to 0.68 USD). The Niger minimum wage equals 30,047 XOF (i.e., 49.98 USD) per month and represents 1137.42 XOF (i.e., 1.89 USD) per day based on 6 working days per week. Thus, the opportunity cost of time for a minimum of 11 h worked (i.e., 60 to 408 XOF) represents ~ 25% daily salary.

In spite of the national policy of exemption from health care costs for pregnant women in Niger [4], the present study showed that most pregnant women (72.5%) experienced some expenses related to ANC. Similarly, previous studies have shown that an exemption of health care costs for pregnant women did not eliminate all costs for women who attended health centers [9–12]. For example, in Rwanda 14.0% of women reported expenses for ANC service [15]. In Niger, the implementation of this policy of exemption from health care costs has faced many challenges. These have included chronic underfunding of this policy, and delays and complex mechanisms of reimbursement for health centers who have provided ANC services free of charge. In addition, there has been opposition among health staff to providing free services mainly because they felt overburdened by the increased number of people who sought care after the implementation of the policy, and the lack of incentive to compensate the health staff’s effort [13]. Finally, a shortage of supplies and essential medications for ANC, has led health staff to issue prescriptions for exams and/or medications instead of providing them for free at the point of care as recommended by the official policy [13, 40].

The majority of women experienced OPC during the first ANC visit and only a small percentage reported OPC in the subsequent visits. Among those who reported expenses during the first visit, nearly all of them were charged either ~ 200 or ~ 300 XOF (i.e., 0.33 or 0.49 USD). Although the present study failed to determine the reason for the expense, it is possible that these costs represented the purchase price of antenatal health booklets.

The present study found that pregnant women who reported spending any money were more likely to have a higher ANC attendance score compared to those who did not, regardless of the scale of ANC attendance score or if the two survey cohorts were considered separately. In contrast, when the data was limited to women that did have to pay for ANC care, mean OPC was associated with a decrease in ANC attendance score based on the eight-recommended ANC contact but this is a relatively small correlation. The results of the sensitivity analyses showed that women who paid any money for a consultation, an exam or medications were more likely to have higher ANC attendance than those who did not. In contrast, previous studies in Lao People’s Democratic Republic and Kenya have shown that OPC (i.e., for service or transportation) negatively affected ANC attendance [41, 42]. The association between any money spent and ANC attendance score in the present study may be due to the fact that those that paid were more able to attend another ANC visit. We found that time spent to attend ANC was not associated with ANC attendance score. This lack of association between time spent and ANC attendance in the present study may be due to the fact that the majority of women reported spending half a day to attend ANC and there was little reported variability among women. Previous studies found inconsistent results regarding the relationship between time spent to attend ANC and ANC attendance. For example, a study conducted in Cambodia revealed that excessive waiting was not associated with low ANC attendance [43]. In contrast, other studies have shown that long waiting time in a health center [43–46] and increased travel time to the health center were associated with low ANC attendance.

In the present study, we did find that women who reported receiving an insecticide-treated bednet or IFA supplements were more likely to have a higher ANC attendance score compared to those who did not, although the receipt of an insecticide-treated bednet was only significantly associated with the ANC score based on four ANC visits. Receiving IFA and possibly insecticide-treated bednets during ANC visits were likely considered an incentive for attending ANC.

The strengths of the present study include an extensive data collection effort that aimed to fill a gap in information regarding OPC, time spent and the opportunity cost of time to attend ANC in rural Niger. However, the study has several limitations. The study was embedded in the NiMaNu project, and thus was not designed specifically with the objectives to examine how OPC and time spent attending ANC are linked with ANC attendance. Because health districts were selected based on convenience sampling, our sample of pregnant women is not representative of the population of the Zinder region nor of Niger. Many women in the present study cohort had not yet completed their third trimester and could therefore not report on costs and time spent that may have occurred in the remainder of their pregnancy. Additional information obtained post-partum would have been helpful. Because the majority of women were illiterate, we had only asked for overall time spent to attend ANC as a categorical variable. Thus, there was a lack of details available for data analyses on time spent to attend ANC, particularly time for transportation and time spent waiting at a health center. Due to the lack of reliable information on participants’ earning and because time spent to attend ANC was reported by women, the opportunity cost of time was estimated based on several assumptions and may have been under or overestimated. Among women who attended ANC, some of them received bednets, medications (e.g., IFA), or food. These items have monetary values that were not included in our analyses. As we mentioned above, the ANC attendance score as presented serves as a proxy on how closely a woman is meeting or not meeting the WHO recommendations on ANC visits. Although it cannot be validated with available data, we have confirmed the sensitivity of this scoring method by comparing the ANC score to the total number of ANC visits, as well as gestational age at the time of interview. It seems to simultaneously capture both of those components better than by adding the numbers of ANC visits to the number of ANC visits completed. For example, the sum of ANC visits is strongly correlated with total number of visits and gestational age at the time of the interview (rho ~ 0.7 for each). The ANC score we have developed is still strongly correlated with total number of ANC visits but not as driven by gestational age at time of interview (rho ~ 0.3). It incorporates relative timing of visit, to indicate whether women are attending visits in the recommended timeframes and also minimizes penalizing women who are earlier in gestation at the time of the interview. In spite of the aforementioned limitations, the present analyses provide important information of ANC attendance and related opportunity costs from the perspective of this previously understudied population.

## Conclusion

The majority of women sought ANC for the first time after their first trimester. The official exemption of health care costs for pregnant women implemented since 2006 in Niger has not eliminated OPCs for pregnant women attending ANC in Zinder. The majority of pregnant women reported OPC, primarily associated with consultation fees, but also including OPC for transportation, medications and exams. However, the present study found that OPCs were associated with ANC attendance score. Consistent associations were found when the eight contact-ANC score was used for this analysis and when the two survey cohorts were considered separately. Women who experienced any OPC were more likely to have a higher ANC attendance score. The majority of women reported spending less than half a day on an ANC visit, and time spent to attend ANC was not associated with the index of overall ANC attendance score. The present study showed that receiving IFA or bednets during any ANC visit were associated with higher ANC attendance scores. These incentives could be used to motivate pregnant women to timely attend ANC. However, such inducements are not costless to policy-makers or to donors, and hence should be set alongside other policy and programmatic options for cost-effectively improving the timing of ANC visit.

## Supplementary Information

**Additional file 1.**

## Data Availability

The datasets generated and analyzed during the current study are available from the study principal investigator on reasonable request (syhess@ucdavis.edu).

## Abbreviations

ANC: Antenatal care
CI: Confidence Interval
GA: Gestational age
HP: Health post
IFA: Iron folic acid
IHC: Integrated Health Center
IQR: Interquartile range
KAP: Knowledge attitude and practice
MDD-W: Minimum dietary diversity for women
Median (Q1, Q3): Median (1st quartile, 3rd quartile)
NiMaNu: Niger Maternal Nutrition
HFIA: Household Food Insecurity Access
SD: Standard deviation
SES: Socio-economic status
OPC: Out-of-pocket costs
USD: United States Dollars
WHO: World Health Organization
XOF: West African CFA

## References

[1] United Nations Inter-agency Group for Child Mortality Estimation (UN IGME) (2018). Levels and trends in child mortality: Report 2018.

[2] Countdown to 2015. A decade of tracking progress for maternal, newborn and child survival. The 2015 report. Available from: http://countdown2030.org/wp-content/uploads/2017/11/Countdown_to_2015_final_report.pdf. (Accessed on 12 September 2018).

[3] Koffi AK, Maina A, Yaroh AG, Habi O, Bensaid K, Kalter HD (2016). Social determinants of child mortality in Niger: results from the 2012 National Verbal and Social Autopsy Study. J Glob Health.

[4] Institut National de la Statistique (2015). Etude sur la gratuité des soins de santé au Niger. Niamey.

[5] Ousseni A (2011). Une politique publique de santé au Niger: la mise en place d’exemptions de paiement des soins en faveur des femmes et des enfants.

[6] World Health Organization (2002). WHO antenatal care randomized trial: manual for the implementation of the new model.

[7] Institut National de la Statistique (INS) & ICF International (2007). Enquête Démographique et de Santé et à Indicateurs Multiples du Niger 2006. Calverton, Maryland, USA.

[8] Institut National de la Statistique & ICF International (2013). Enquête Démographique et de Santé et à Indicateurs Multiples du Niger 2012. Calverton, Maryland, USA.

[9] Diarra A (2012). Mise en œuvre locale de l’exemption des paiements des soins au Niger: évaluation dans les districts sanitaires. Afr Contemp.

[10] Dalinjong PA, Wang AY, Homer CSE (2018). Has the free maternal health policy eliminated out of pocket payments for maternal health services? Views of women, health providers and insurance managers in Northern Ghana. PLoS One.

[11] Rahman M, Rob U, Noor FR, Bellows B (2012). Out-of-pocket expenses for maternity care in rural Bangladesh: a public-private comparison. Int Q Community Health Educ.

[12] Leone T, James KS, Padmadas SS (2013). The burden of maternal health care expenditure in India: multilevel analysis of national data. Matern Child Health J.

[13] Olivier de Sardan JP, Ridde V (2012). L’exemption de paiment des soins au Burkina Faso, Mali et Niger: les contractions des politiques publiques. Afr Contemp.

[14] Verhoef TI, Daley R, Vallejo-Torres L, Chitty LS, Morris S (2016). Time and travel costs incurred by women attending antenatal tests: a costing study. Midwifery.

[15] Hitimana R, Lindholm L, Krantz G, Nzayirambaho M, Pulkki-Brannstrom AM (2018). Cost of antenatal care for the health sector and for households in Rwanda. BMC Health Serv Res.

[16] Simkhada B, Teijlingen ER, Porter M, Simkhada P (2008). Factors affecting the utilization of antenatal care in developing countries: systematic review of the literature. J Adv Nurs.

[17] Banke-Thomas OE, Banke-Thomas AO, Ameh CA (2017). Factors influencing utilisation of maternal health services by adolescent mothers in Low-and middle-income countries: a systematic review. BMC Pregnancy Childbirth.

[18] Ali SA, Dero AA, Ali SA, Ali GB. Factors affecting the utilization of antenatal care among pregnant women: A literature review. J Preg Neonatal Med. 2(2):41–5.

[19] Kruk ME, Paczkowski M, Mbaruku G, de Pinho H, Galea S. Women’s preferences for place of delivery in rural Tanzania: a population-based discrete choice experiment. Am J Public Health 2009;99(9):1666–1672. 10.2105/AJPH.2008.146209 PMC2724466 19608959

[20] Kruk ME, Paczkowski MM, Tegegn A, Tessema F, Hadley C, Asefa M (2010). Women’s preferences for obstetric care in rural Ethiopia: a population-based discrete choice experiment in a region with low rates of facility delivery. J Epidemiol Community Health.

[21] Hess SY, Ouedraogo CT, Bamba IF, Wessells KR, Keith N, Faye T (2018). Using formative research to promote antenatal care attendance and iron folic acid supplementation in Zinder, Niger. Matern Child Nutr.

[22] Begum K, Ouedraogo CT, Wessells KR, Young RR, Faye MT, Wuehler SE, et al. Prevalence of and factors associated with antenatal care seeking and adherence to recommended iron-folic acid supplementation among pregnant women in Zinder, Niger. Matern Child Nutr. 2018;14(Suppl 1). 10.1111/mcn.12466. 10.1111/mcn.12466 PMC6866102 29493896

[23] Wessells KR, Ouedraogo CT, Young RR, Faye MT, Brito A, Hess SY. Micronutrient status among pregnant women in Zinder, Niger and risk factors associated with deficiency. Nutrients. 2017;9(5). 10.3390/nu9050430. 10.3390/nu9050430 PMC5452160 28445440

[24] Ouedraogo CT, Wessells KR, Young RR, Bamba IF, Faye MT, Banda N (2019). The mixed effects of a package of multilevel interventions on the health and care of pregnant women in Zinder, Niger. BMJ Glob Health.

[25] Ridde V, Diarra A (2009). A process evaluation of user fees abolition for pregnant women and children under five years in two districts in Niger (West Africa). BMC Health Serv Res.

[26] Institut National de la Statistique (2013). Manuel des concepts et definitions. Niamey.

[27] United Nations (2008). Designing household survey samples: pratical guidelines. New York.

[28] Hess SY, Ouedraogo CT (2016). NiMaNu project. Open Science Framework.

[29] Pound Sterline Live (2015). Historical rates for the USD/XOF currency conversion on 31 December 2015.

[30] World Vision (2015). Moringa improves livelihoods and nutrition in Niger.

[31] Moussa S (2011). Impact de l’élevage de la chèvre rousse de Maradi sur le statut socio-economique de la femme rurale. Master d’Economie et Politique d’Elevage. Université Cheick Anta Diop de Dakar.

[32] Vyas S, Kumaranayake L (2006). Constructing socio-economic status indices: how to use principal components analysis. Health Policy Plan.

[33] Coates J, Swindale A, Bilinsky P (2007). Household Food Insecurity Access Scale (HFIAS) for Measurement of Household Food Access: Indicator Guide (v.3). Washington, DC.

[34] FAO and FHI 360 (2016). Minimum Dietary Diversity for Women: a guide for the measurement. Rome.

[35] Moller AB, Petzold M, Chou D, Say L (2017). Early antenatal care visit: a systematic analysis of regional and global levels and trends of coverage from 1990 to 2013. Lancet Glob Health.

[36] République du Niger (2017). Ministère de la Santé Publique, Direction des Statistiques. Annuaire des Statistiques Sanitaires du Niger 2016.

[37] Lule GS, Tugumisirize J, Ndekha M (2000). Quality of care and its effects on utilisation of maternity services at health centre level. East Afr Med J.

[38] République du Niger (2014). Ministère de la Santé Publique, Direction des Statistiques. Annuaire des Statistiques Sanitaires du Niger 2013.

[39] République du Niger (2015). Ministère de la Santé Publique, Direction des Statistiques. Annuaire des Statistiques Sanitaires du Niger 2015.

[40] Diarra A, Ousseini A (2015). The coping strategies of front-line health workers in the context of user fee exemptions in Niger. BMC Health Serv Res.

[41] Ye Y, Yoshida Y, Harun-Or-Rashid M, Sakamoto J (2010). Factors affecting the utilization of antenatal care services among women in Kham District, Xiengkhouang province. Lao PDR. Nagoya J Med Sci.

[42] van Eijk AM, Bles HM, Odhiambo F, Ayisi JG, Blokland IE, Rosen DH (2006). Use of antenatal services and delivery care among women in rural western Kenya: a community based survey. Reprod Health.

[43] Zafar AA, Ehiri JE, Anyanwu EC (2003). Use of antenatal services in Kampung District, Cambodia. Sci World J.

[44] Mason L, Dellicour S, Ter Kuile F, Ouma P, Phillips-Howard P, Were F (2015). Barriers and facilitators to antenatal and delivery care in western Kenya: a qualitative study. BMC Pregnancy Childbirth.

[45] Ganga-Limando M, Gule WP (2015). Potential barriers to focused antenatal care utilisation by HIV-positive pregnant women in Swaziland. South Afr Fam Pract.

[46] Kruk ME, Mbaruku G, Rockers PC, Galea S (2008). User fee exemptions are not enough: out-of-pocket payments for ‘free’ delivery services in rural Tanzania. Trop Med Int Health.

