# Supplementary material for: Out-of-pocket costs and time spent attending antenatal care services: a case study of pregnant women in selected rural communities in Zinder, Niger
Source: BMC Health Serv Res. 2021 Jan 8;21:47. doi: 10.1186/s12913-020-06027-2 (PMC7796614; doi:10.1186/s12913-020-06027-2)
Supplement: Supplementary file 1 — Additional file 1. [file 12913_2020_6027_MOESM1_ESM.docx]

**Supplemental Table 1**: Characteristics of pregnant women in the baseline vs endline surveys^1, 2^

|  | **Baseline** | **Endline** | **P-value** |
| --- | --- | --- | --- |
| **N** | **1385** | **922** |  |
| Adolescent (≤19 years) | 221 (16.3%) | 197 (21.5%) |  |
| Adult (>19 years) | 1131 (83.7%) | 718 (78.5) | 0.002 |
| **Ethnicity** |  |  |  |
| Hausa | 1188 (85.8%) | 763 (82.9%) |  |
| Others | 196 (14.2%) | 157 (17.1%) | 0.06 |
| **Education** |  |  |  |
| Koranic school, only literacy training or no education | 1095 (79.1%) | 723 (78.6%) |  |
| Some primary education or higher | 289 (20.9%) | 197 (21.4) | 0.76 |
| **Principal occupation** |  |  |  |
| Housewife | 1151 (83.2%) | 745 (80.9%) |  |
| Non-Housewife | 233 (16.8%) | 176 (19.1%) | 0.16 |
| **Marital status^3^** |  |  |  |
| Separated/divorced or widowed | 13 (0.9%) | 8.0 (0.9%) |  |
| Married | 1363 (99.1%) | 913 (99.1%) | - |
| **Trimester** |  |  |  |
| First or second | 467 (39.5%) | 324 (38.3%) |  |
| Third | 714 (60.5%) | 522 (61.7%) | 0.57 |
| **Obstetric history** |  |  |  |
| Age at first pregnancy, median (25^th^,75^th^)  (min- max) | 16.0 (16.0, 17.0)  (12.0 - 35.0) | 16.0 (16.0, 18.0)  (11.0 - 38.0) |  |
| **Gravidity** |  |  |  |
| Primigravida | 178 (12.9%) | 139 (15.1%) |  |
| Multigravida | 1206 (87.1%) | 782 (84.9%) | 0.13 |

| **Outcome of previous pregnancy** |  |  |  |
| --- | --- | --- | --- |
| Child born alive, still living | 1029 (85.3 %) | 685 (87.6%) |  |
| Child not born alive or born alive and has since died | 177 (14.7%) | 97 (12.4%) | 0.27 |
| **Attended any ANC during last pregnancy** | 1108 (91.9%) | 727 (93.0%) | 0.37 |
| **Attended at least 4 ANC during last pregnancy** | 476 (39.5%) | 300 (38.4%) | 0.62 |
| **Health facility delivery during last pregnancy** | 419 (34.9%) | 310 (39.7%) | 0.03 |
| **Household level characteristics** |  |  |  |
| **Household head’s education level** |  |  |  |
| No education or literacy training only or koranic school | 950 (78.4%) | 635 (81.7%) |  |
| Some primary education or higher | 261 (21.6%) | 142 (18.3%) | 0.08 |
| **Principal occupation of the household head** |  |  |  |
| Farming related occupation | 578 (42.0%) | 205 (22.6%) |  |
| Non-farming related occupation | 798 (58.0%) | 700 (77.4%) | <.0001 |
| **Levels of household food insecurity** |  |  |  |
| Food secure | 432 (31.3%) | 557 (60.5%) |  |
| Mildly food insecure | 150 (10.8%) | 52 (5.6%) |  |
| Moderately food insecure | 345 (25.0%) | 185 (20.1%) |  |
| Severely food insecure | 454 (32.9%) | 127 (13.8%) | <.0001 |
| **Season of enrollment** |  |  |  |
| Hot (March-May) | 416 (30.1%) | 302 (32.8%) |  |
| Raining (June-September) | 501 (36.2%) | 275 (29.9%) |  |
| Dry (October-February) | 467 (33.7%) | 344 (37.3%) | 0.007 |

^1^ANC, antenatal care; min, minimum; max, maximum; ^2^Results are presented as N (%) or mean ± SD, unless stated otherwise; ^3^Categories were too small to calculate a p-value

**Supplemental Table 2a**: ANC attendance score based on 4 ANC visits by gestational age (trimester) of pregnancy for baseline cohort

|  | **First trimester** | **Second trimester** | **First half of third trimester** | **Second half of third trimester** |
| --- | --- | --- | --- | --- |
| Minimum acceptable ANC attendance score | 0 | 1 | 2 | 3 |
| Maximum possible ANC attendance score | 1 | 2 | 3 | 4 |
| Median (Q1, Q3) attendance score^c^ | 0 (-1, 1) | 0 (-1, 0) | 0 (-1, 1) | 1 (-1, 1) |

^a^ANC = Antenatal care;

^b^Trimesters were defined as: first trimester, ≤13 weeks; second trimester: >13weeks to 27 weeks; first half of third trimester: >27 weeks day to 34
weeks; and second half of third trimester: >34weeks

^c^Median (1^st^ quartile (Q1), 3^rd^ quartile (Q3))

**Supplemental Table 2b**: ANC attendance score based on 4 ANC visits by gestational age (trimester) of pregnancy for endline cohort

|  | **First trimester** | **Second trimester** | **First half of third trimester** | **Second half of third trimester** |
| --- | --- | --- | --- | --- |
| Minimum acceptable ANC attendance score | 0 | 1 | 2 | 3 |
| Maximum possible ANC attendance score | 1 | 2 | 3 | 4 |
| Median (Q1, Q3) attendance score^c^ | -0.5 (-1, 0) | 0 (-1, 0) | -1 (-1, 1) | 1 (-1, 1) |

^a^ANC = Antenatal care;

^b^Trimesters were defined as: first trimester, ≤13 weeks; second trimester: >13weeks to 27 weeks; first half of third trimester: >27 weeks day to 34
weeks; and second half of third trimester: >34weeks

^c^Median (1^st^ quartile (Q1), 3^rd^ quartile (Q3))

**Supplemental Table 3a**: ANC attendance score based on eight ANC contacts by gestational age (trimester) of pregnancy^a, b^

|  | **1^st^ trimester** | **1^st^ half of 2^nd^ trimester** | **2^nd^ half of 2^nd^ trimester** | **1/5 of 3^rd^ trimester** | **2/5 of 3^rd^ trimester** | **3/5 of 3^rd^ trimester** | **4/5 of 3^rd^ trimester** | **5/5 of 3^rd^ trimester** |
| --- | --- | --- | --- | --- | --- | --- | --- | --- |
| Minimum acceptable ANC attendance score | 0 | 1 | 2 | 3 | 4 | 5 | 6 | 7 |
| Maximum possible ANC attendance score | 1 | 2 | 3 | 4 | 5 | 6 | 7 | 8 |
| Median (Q1, Q3) attendance score^c^ | 0 (-1, 0) | 0 (-1, 0) | -1 (-1, -1) | -1 (-2, 0) | -1 (-2, 1) | -1 (-1, 1) | 0 (-2, 0) | 1 (-0.5, 2) |

^a^ANC = Antenatal care;

^b^Trimesters were defined as: first trimester, ≤13 weeks; first half of second trimester: >13weeks to 21 weeks; second half of second trimester: >21 weeks day to 27 weeks; first fifth of third trimester: >27weeks day to 31 week; second fifth of third trimester: >31weeks day to 35 weeks; third fifth of third trimester: >35 weeks day to 37 weeks; fourth fifth of third trimester: >37weeks day to 39 weeks; last fifth of third trimester: >39weeks

^c^Median (1^st^ quartile (Q1), 3^rd^ quartile (Q3))

**Supplemental Table 3b**: ANC attendance score based on eight ANC contacts by gestational age (trimester) of pregnancy for baseline cohort

|  | **1^st^ trimester** | **1^st^ half of 2^nd^ trimester** | **2^nd^ half of 2^nd^ trimester** | **1/5 of 3^rd^ trimester** | **2/5 of 3^rd^ trimester** | **3/5 of 3^rd^ trimester** | **4/5 of 3^rd^ trimester** | **5/5 of 3^rd^ trimester** |
| --- | --- | --- | --- | --- | --- | --- | --- | --- |
| Minimum acceptable ANC attendance score | 0 | 1 | 2 | 3 | 4 | 5 | 6 | 7 |
| Maximum possible ANC attendance score | 1 | 2 | 3 | 4 | 5 | 6 | 7 | 8 |
| Median (Q1, Q3) attendance score^c^ | 0 (-1, 1) | 0 (-1, 0) | -1 (-1.5, -1) | -1 (-2, 1) | 0 (-2, 1) | -1 (-1, 1) | 0 (-2, 0) | 0.5 (-0.5, 1.5) |

^a^ANC = Antenatal care;

^b^Trimesters were defined as: first trimester, ≤13 weeks; first half of second trimester: >13weeks to 21 weeks; second half of second trimester: >21 weeks day to 27 weeks; first fifth of third trimester: >27weeks day to 31 week; second fifth of third trimester: >31weeks day to 35 weeks; third fifth of third trimester: >35 weeks day to 37 weeks; fourth fifth of third trimester: >37weeks day to 39 weeks; last fifth of third trimester: >39weeks

^c^Median (1^st^ quartile (Q1), 3^rd^ quartile (Q3))

**Supplemental Table 3c**: ANC attendance score based on eight ANC contacts by gestational age (trimester) of pregnancy for endline cohort

|  | **1^st^ trimester** | **1^st^ half of 2^nd^ trimester** | **2^nd^ half of 2^nd^ trimester** | **1/5 of 3^rd^ trimester** | **2/5 of 3^rd^ trimester** | **3/5 of 3^rd^ trimester** | **4/5 of 3^rd^ trimester** | **5/5 of 3^rd^ trimester** |
| --- | --- | --- | --- | --- | --- | --- | --- | --- |
| Minimum acceptable ANC attendance score | 0 | 1 | 2 | 3 | 4 | 5 | 6 | 7 |
| Maximum possible ANC attendance score | 1 | 2 | 3 | 4 | 5 | 6 | 7 | 8 |
| Median (Q1, Q3) attendance score^c^ | 0.5 (-1, 0) | 0 (-1, 0) | -1 (-1, 0) | -1 (-2, 0) | -1 (-2, 0) | -1 (-1, 1) | 0 (-2, 0) | 1.5 (-1, 2) |

^a^ANC = Antenatal care;

^b^Trimesters were defined as: first trimester, ≤13 weeks; first half of second trimester: >13weeks to 21 weeks; second half of second trimester: >21 weeks day to 27 weeks; first fifth of third trimester: >27weeks day to 31 week; second fifth of third trimester: >31weeks day to 35 weeks; third fifth of third trimester: >35 weeks day to 37 weeks; fourth fifth of third trimester: >37weeks day to 39 weeks; last fifth of third trimester: >39weeks

^c^Median (1^st^ quartile (Q1), 3^rd^ quartile (Q3))

**Supplemental Table 4**: Maternal and household characteristics by survey cohort associated with ANC attendance score based on four ANC visits^a,b,c^

|  | **Baseline sample**  **n=1023** | | **Endline sample**  **n=713** | | | | | | | |
| --- | --- | --- | --- | --- | --- | --- | --- | --- | --- | --- |
|  | **Spearman correlation or**  **mean difference**  **(95% CI)** | **P-value^e^** | **Spearman correlation or mean difference (95% CI)** |  |  |  |  |  |  | **P-value^e^** |
| **Age, years** | 0.05 | 0.09 | 0.02 |  |  |  |  |  |  | 0.61 |
| **Adolescent** |  |  |  |  |  |  |  |  |  |  |
| Yes | -0.02 (-0.18, 0.15) | 0.84 | -0.17 (-0.35, 0.01) |  |  |  |  |  |  | 0.07 |
| No | Ref |  | Ref |  |  |  |  |  |  |  |
| **Education level** |  |  |  |  |  |  |  |  |  |  |
| Any formal education | -0.03 (-0.18, 0.12) | 0.69 | 0.05 (-0.13, 0.23) |  |  |  |  |  |  | 0.60 |
| No education | Ref |  | Ref |  |  |  |  |  |  |  |
| **Rank of woman in polygamous marriage** | 0.04 | 0.47 | 0.02 |  |  |  |  |  |  | 0.80 |
| **Principal occupation** |  |  |  |  |  |  |  |  |  |  |
| Housewife | -0.04 (-0.20, 0.12) | 0.63 | 0.06 (-0.12, 0.25) |  |  |  |  |  |  | 0.51 |
| Non-housewife | Ref |  | Ref |  |  |  |  |  |  |  |
| **Age at first pregnancy** | 0.02 | 0.58 | -0.004 |  |  |  |  |  |  | 0.90 |
| **Number of pregnancies** | 0.04 | 0.29 | 0.01 |  |  |  |  |  |  | 0.83 |
| **Number of living children** | 0.06 | 0.06 | 0.001 |  |  |  |  |  |  | 0.98 |
| **Outcome of previous pregnancy** |  |  |  |  |  |  |  |  |  |  |
| Child born alive, still living | 0.004 (-0.18, 0.19) | 0.96 | -0.11 (-0.36, 0.14) |  |  |  |  |  |  | 0.37 |
| Child not born alive or born alive   and has since died | Ref |  | Ref |  |  |  |  |  |  |  |
| **Health facility delivery in previous pregnancy** | 0.28 (0.15, 0.42) | <0.0001 | -0.04 (-0.21, 0.12) |  |  |  |  |  |  | 0.60 |
| **Gestational age (weeks)** | 0.34 | <0.0001 | 0.31 |  |  |  |  |  |  | <0.0001 |
| **Reported experiencing any danger signs in current pregnancy** | -0.11 (-0.37, 0.15) | 0.40 | 0.20 (-0.11, 0.52) |  |  |  |  |  |  | 0.20 |
| **Referred to health center because of undernutrition at visit 1^d^** | -0.10 (-0.24, 0.04) | 0.16 | 0.007 (-0.11, 0.25) |  |  |  |  |  |  | 0.44 |
| **Reported receiving food assistance the month before the interview** | 0.34 (0.13, 0.54) | 0.001 | 0.33 (0.11, 0.55) |  |  |  |  |  |  | 0.003 |
| **Received mosquito net** | 0.29 (0.11, 0.48) | 0.002 | 0.09 (-0.09, 0.26) |  |  |  |  |  |  | 0.33 |
| **Has received iron folic acid supplements** | 0.65 (0.53, 0.77) | <0.0001 | 0.89 (0.72, 1.06) |  |  |  |  |  |  | <0.0001 |
| **Household asset index** |  |  |  |  |  |  |  |  |  |  |
| Above median | 0.19 (0.06, 0.31) | 0.003 | 0.21 (0.05, 0.36) |  |  |  |  |  |  | 0.008 |
| At or below the median | Ref |  | Ref |  |  |  |  |  |  |  |
| **Household livestock index** |  |  |  |  |  |  |  |  |  |  |
| Above median | -0.02 (-0.14, 0.10) | 0.80 | 0.02 (-0.13, 0.17) |  |  |  |  |  |  | 0.82 |
| At or below the median | Ref |  | Ref |  |  |  |  |  |  |  |
| **Housing quality** |  |  |  |  |  |  |  |  |  |  |
| Above median | 0.13 (-0.01, 0.26) | 0.06 | 0.09 (-0.06, 0.25) |  |  |  |  |  |  | 0.24 |
| At or below the median | Ref |  | Ref |  |  |  |  |  |  |  |
| **Household head’s education level** |  |  |  |  |  |  |  |  |  |  |
| Any formal education | 0.12 (-0.03, 0.28) | 0.12 | 0.20 (-0.006, 0.40) |  |  |  |  |  |  | 0.06 |
| No education | Ref |  | Ref |  |  |  |  |  |  |  |
| **Principal occupation of the household head** |  |  |  |  |  |  |  |  |  |  |
| Farming related occupation | -0.18 (-0.30, -0.05) | 0.006 | -0.03 (-0.22, 0.15) |  |  |  |  |  |  | 0.74 |
| Non-farming related occupation | Ref |  | Ref |  |  |  |  |  |  |  |
| **Levels of household food insecurity** |  |  |  |  |  |  |  |  |  |  |
| Food secure | 0.13 (-0.02, 0.29) | 0.22 | 0.06 (-0.17, 0.29) |  |  |  |  |  |  | 0.19 |
| Mildly food insecure | 0.08 (-0.13, 0.30) |  | 0.36 (-0.01, 0.73) |  |  |  |  |  |  |  |
| Moderately food insecure | 0.15 (-0.01, 0.32) |  | -0.01 ( -0.28, 0.26) |  |  |  |  |  |  |  |
| Severely food insecure | Ref |  | Ref |  |  |  |  |  |  |  |
| **Season of enrollment** |  |  |  |  |  |  |  |  |  |  |
| Lean, rain (June-September) | -0.21 (-0.36, -0.06) | 0.02 | -0.03 (-0.22, 0.16) |  |  |  |  |  |  | 0.73 |
| Dry, post-harvest (October-February) | -0.09 (-0.24, 0.06) |  | -0.07 (-0.25, 0.11) |  |  |  |  |  |  |  |
| Hot (March-May) | Ref |  | Ref |  |  |  |  |  |  |  |

^a^ANC, Antenatal care
 ^b^Only women who attended ANC visits

^c^P-values are from Spearman correlation (for continuous variable) and t-test/omnibus ANOVA F-test
 (for categorical variables); R: significance at 0.10
 ^d^Undernutrition defined as mid-upper arm circumference (MUAC) < 23 cm

**Supplemental Table 5**: Maternal and household characteristics by survey cohort associated with ANC attendance score based on eight ANC contacts^a,b,c^

|  | **Baseline sample**  **n=1023** | | **Endline sample**  **n=713** | | | | | | | |
| --- | --- | --- | --- | --- | --- | --- | --- | --- | --- | --- |
|  | **Spearman correlation or**  **mean difference**  **(95% CI)** | **P-value^e^** | **Spearman correlation or mean difference (95% CI)** |  |  |  |  |  |  | **P-value^e^** |
| **Age, years** | -0.004 | 0.90 | 0.01 |  |  |  |  |  |  | 0.77 |
| **Adolescent** |  |  |  |  |  |  |  |  |  |  |
| Yes | 0.06 (-0.21, 0.33) | 0.66 | -0.21 (-0.48, 0.06) |  |  |  |  |  |  | 0.13 |
| No | Ref |  | Ref |  |  |  |  |  |  |  |
| **Education level** |  |  |  |  |  |  |  |  |  |  |
| Any formal education | -0.02 (-0.26, 0.22) | 0.84 | 0.07 (-0.21, 0.34) |  |  |  |  |  |  | 0.63 |
| No education | Ref |  | Ref |  |  |  |  |  |  |  |
| **Rank of woman in polygamous marriage** | 0.06 | 0.29 | -0.01 |  |  |  |  |  |  | 0.84 |
| **Principal occupation** |  |  |  |  |  |  |  |  |  |  |
| Housewife | -0.20 (-0.46, 0.07) | 0.14 | -014 (-0.43, 0.14) |  |  |  |  |  |  | 0.33 |
| Non-housewife | Ref |  | Ref |  |  |  |  |  |  |  |
| **Age at first pregnancy** | 0.01 | 0.75 | 0.01 |  |  |  |  |  |  | 0.76 |
| **Number of pregnancies** | 0.04 | 0.29 | -0.06 |  |  |  |  |  |  | 0.12 |
| **Number of living children** | 0.05 | 0.17 | -0.05 |  |  |  |  |  |  | 0.23 |
| **Outcome of previous pregnancy** |  |  |  |  |  |  |  |  |  |  |
| Child born alive, still living | 0.002 (-0.30, 0.30) | 0.99 | -0.35 (-0.73, 0.03) |  |  |  |  |  |  | 0.07 |
| Child not born alive or born alive   and has since died | Ref |  | Ref |  |  |  |  |  |  |  |
| **Health facility delivery in previous pregnancy** | 042 (0.21, 0.64) | 0.0001 | 0.08 (-0.17, 0.33) |  |  |  |  |  |  | 0.52 |
| **Gestational age (weeks)** | 0.06 | 0.06 | -0.003 |  |  |  |  |  |  | 0.94 |
| **Reported experiencing any danger signs in current pregnancy** | -0.05 (-0.47, 0.37) | 0.81 | 0.06 (-0.41, 0.53) |  |  |  |  |  |  | 0.80 |
| **Referred to health center because of undernutrition at visit 1^d^** | -0.02 (-0.25, 0.20) | 0.83 | 0.15 (-0.12, 0.41) |  |  |  |  |  |  | 0.27 |
| **Reported receiving food assistance the month before the interview** | 0.53 (0.20, 0.86) | 0.002 | 0.41 (0.08, 0.75) |  |  |  |  |  |  | 0.01 |
| **Received mosquito net** | 0.22 (-0.08, 0.52) | 0.16 | -0.14 (-0.40, 0.12) |  |  |  |  |  |  | 0.29 |
| **Has received iron folic acid supplements** | 0.82 (0.62, 1.02) | <0.0001 | 1.04 (0.78, 1.30) |  |  |  |  |  |  | <0.0001 |
| **Household asset index** |  |  |  |  |  |  |  |  |  |  |
| Above median | 0.21 (0.01, 0.41) | 0.03 | 0.24 (0.01, 0.48) |  |  |  |  |  |  | 0.04 |
| At or below the median | Ref |  | Ref |  |  |  |  |  |  |  |
| **Household livestock index** |  |  |  |  |  |  |  |  |  |  |
| Above median | 0.02 (-01.8, 022) | 0.86 | 0.06 (-0.16, 0.29) |  |  |  |  |  |  | 0.57 |
| At or below the median | Ref |  | Ref |  |  |  |  |  |  |  |
| **Housing quality** |  |  |  |  |  |  |  |  |  |  |
| Above median | 0.21 (-0.01, 0.43) | 0.06 | -0.07 (-0.30, 0.17) |  |  |  |  |  |  | 0.57 |
| At or below the median | Ref |  | Ref |  |  |  |  |  |  |  |
| **Household head’s education level** |  |  |  |  |  |  |  |  |  |  |
| Any formal education | 0.18 (-0.07, 0.43) | 0.16 | 0.29 (-0.02, 0.60) |  |  |  |  |  |  | 0.07 |
| No education | Ref |  | Ref |  |  |  |  |  |  |  |
| **Principal occupation of the household head** |  |  |  |  |  |  |  |  |  |  |
| Farming related occupation | -0.38 (-0.58, -0.18) | 0.0002 | -0.01 (-0.29, 0.28) |  |  |  |  |  |  | 0.96 |
| Non-farming related occupation | Ref |  | Ref |  |  |  |  |  |  |  |
| **Levels of household food insecurity** |  |  |  |  |  |  |  |  |  |  |
| Food secure | 0.27 (0.02, 0.52) | 0.11 | 0.004 (-0.34, 0.35) |  |  |  |  |  |  | 0.99 |
| Mildly food insecure | 0.15 (-0.20, 0.50) |  | -0.03 (-0.59, 0.53) |  |  |  |  |  |  |  |
| Moderately food insecure | 0.28 (0.01, 0.54) |  | -0.05 (-0.46, 0.36) |  |  |  |  |  |  |  |
| Severely food insecure | Ref |  | Ref |  |  |  |  |  |  |  |
| **Season of enrollment** |  |  |  |  |  |  |  |  |  |  |
| Lean, rain (June-September) | -0.34 (-0.58, -0.10) | 0.02 | -0.21 (-0.50, 0.07) |  |  |  |  |  |  | 0.25 |
| Dry, post-harvest (October-February) | -0.13 (-0.38, 0.11) |  | -0.20 (-0.47, 0.07) |  |  |  |  |  |  |  |
| Hot (March-May) | Ref |  | Ref |  |  |  |  |  |  |  |

^a^ANC, Antenatal care
 ^b^Only women who attended ANC visits

^c^P-values are from Spearman correlation (for continuous variable) and t-test/omnibus ANOVA F-test
 (for categorical variables); R: significance at 0.10
 ^d^Undernutrition defined as mid-upper arm circumference (MUAC) < 23 cm

**Supplemental Table 6**: Association between out-of-pocket costs and time cost of attending ANC visit and ANC attendance score based on the 2002 WHO recommendation of at least four ANC visits among pregnant women in the baseline and the endline survey

|  | **ANC attendance score based on four ANC visits in baseline survey  (n=1023)** | | | | **ANC attendance score based on four ANC visits in endline survey (n=713)** | | | |
| --- | --- | --- | --- | --- | --- | --- | --- | --- |
|  | **Minimally adjusted Spearman correlation or mean difference**  **(95% CI)^a^** | **P-value** | **Adjusted Spearman correlation or mean difference**  **(95% CI)^b^** | **P-value** | **Minimally adjusted Spearman correlation or mean difference**  **(95% CI)^a^** | **P-value** | **Adjusted Spearman correlation or mean difference**  **(95% CI)^b^** | **P-value** |
| **Any out-of-pocket costs spent per ANC visit** |  |  |  |  |  |  |  |  |
| Yes | 0.34 (0.19, 0.48) | <0.0001 | 0.29 (0.13, 0.46) | 0.0006 | 0.25 (0.08, 0.42) | 0.004 | 0.12 (-0.06, 0.30) | 0.18 |
| No | Ref |  | Ref |  | Ref |  | Ref |  |
| **Mean out-of-pocket costs (XOF)^d^** | -0.01 | 0.72 | -0.03 | 0.44 | 0.005 | 0.90 | -0.05 | 0.22 |
| **Mean time spent (hours)** | 0.02 | 0.51 | 0.05 | 0.19 | 0.008 | 0.82 | 0.03 | 0.42 |

^a^Controlling for only for gestational age (in week); ^b^Controlling for only variables significantly associated with the outcomes at a level of significance of 0.1 in bivariate analysis

**Supplemental Table 7**: Association between out-of-pocket costs and time cost of attending ANC visit and ANC attendance score based on the 2016 WHO recommendation of at least eight ANC visits among pregnant women in the baseline and the endline survey

|  | **ANC attendance score based on four ANC visits in baseline survey  (n=1023)** | | | | **ANC attendance score based on four ANC visits in endline survey  (n=713)** | | | |
| --- | --- | --- | --- | --- | --- | --- | --- | --- |
|  | **Minimally adjusted Spearman correlation or mean difference**  **(95% CI)^a^** | **P-value** | **Adjusted Spearman correlation or mean difference**  **(95% CI)^b^** | **P-value** | **Minimally adjusted Spearman correlation or mean difference**  **(95% CI)^a^** | **P-value** | **Adjusted Spearman correlation or mean difference**  **(95% CI)^b^** | **P-value** |
| **Any out-of-pocket costs spent per ANC visit** |  |  |  |  |  |  |  |  |
| Yes | 0.45 (0.18, 0.71) | 0.0009 | 0.50 (0.22, 0.78) | 0.0004 | 0.06 (-0.20, 0.3) | 0.36 | 0.16 (-0.40, 0.24) | 0.62 |
| No | Ref |  | Ref |  | Ref |  | Ref |  |
| **Mean out-of-pocket costs (XOF)^d^** | -0.06 | 0.04 | -0.08 | 0.02 | -0.10 | 0.01 | -0.16 | 0.0006 |
| **Mean time spent (hours)** | -0.009 | 0.77 | 0.008 | 0.81 | 0.001 | 0.97 | 0.07 | 0.13 |

^a^Controlling for only for gestational age (in week); ^b^Controlling for only variables significantly associated with the outcomes at a level of significance of 0.1 in bivariate analysis
